# Reliability Generalization Study of the Person-Centered Care Assessment Tool

**DOI:** 10.3389/fpsyg.2021.712582

**Published:** 2021-09-27

**Authors:** Lluna María Bru-Luna, Manuel Martí-Vilar, César Merino-Soto, José Livia

**Affiliations:** ^1^Departamento de Psicología Básica, Universitat de València, Valencia, Spain; ^2^Instituto de Investigación de Psicología, Universidad de San Martín de Porres, Chiclayo, Peru; ^3^Universidad Nacional Federico Villareal, Lima, Peru

**Keywords:** reliability generalization meta-analysis, assessment, person-centered care assessment tool, person-centered care (PCC), measurement

## Abstract

The so-called Person-Centered Care (PCC) model identifies three fundamental principles: changing the focus of attention from the disease to the person, individualizing care, and promoting empowerment. The Person-Centered Care Assessment Tool (P-CAT) has gained wide acceptance as a measure of PCC in recent years due to its brevity and simplicity, as well as its ease of application and interpretation. The objective of this study is to carry out a reliability generalization meta-analysis to estimate the internal consistency of the P-CAT and analyze possible factors that may affect it, such as the year of publication, the care context, the application method, and certain sociodemographic properties of the study sample. The mean value of α for the 25 samples of the 23 studies in the meta-analysis was 0.81 (95% CI: 0.79–0.84), with high heterogeneity (squared-*I* = 85.83%). The only variable that had a statistically significant relationship with the reliability coefficient was the mean age of the sample. The results show that the P-CAT gives acceptably consistent scores when its use is oriented toward the description and investigation of groups, although it may be affected by variables such as the age of participants.

## Reliability Generalization Meta-Analysis of the Person-Centered Care Assessment Tool

More and more people require care and support of different types and intensity. The traditional model of care that currently prevails makes it impossible for these people to develop life plans and maintain control of their lives both in long-term decisions, such as where and with whom to live or what type of treatment to receive, and in everyday aspects through the imposition of schedules for getting up, eating and leisure activities (Rodríguez, 2013). There is a growing demand for care plans to include objectives that go beyond treating illnesses and/or reducing the situation of dependency. In most European countries, these formal long-term care systems combine economic benefits, residential care, and home services; but other types of services are much less common, such as those that promote personal autonomy, counseling, guidance, and case management (Zalakain, 2017). In the traditional model of care, the user has to adjust to a system focused on attention and problem-solving, where professionals and organizations set the guidelines, and in which the subject has a passive role as a mere recipient of services. It is thus important to highlight the efforts being made in various countries to move toward a new paradigm of care, characterized by aspects such as deinstitutionalization, quality of life, and person-centered care, among others (Zalakain, 2017).

The so-called Person-Centered Care (PCC) model was first described within the psychotherapy of Rogers (1961), whose Client-Centered Therapy was based on the psychotherapist's deep attitudes of respect and acceptance toward the client and the latter's capacities for change. Rogers's proposals have been transferred to different fields of intervention such as education, medicine, geriatrics, and functional diversity (Martínez, 2013). The PCC identifies three aspects of care as fundamental principles (Smith and Williams, 2016): the change of the focus of attention of the disease to the person (i.e., taking into account the experiences and values of each individual), individualized care (determined by the needs and preferences of each person rather than by the standards of the organization) and the promotion of empowerment (i.e., respecting the patient's values and freedom of choice).

Although the use of the term PCC has become increasingly common in health and social care services around the world (McCormack et al., 2015), there is a lack of consensus and clear definition regarding its meaning and the processes involved in its application, which can become a barrier for both implementation and evaluation of PCC (Rathert et al., 2013; Sharma et al., 2016). For example, other components identified for the practice of PCC include autonomy, individuality, intimacy, independence, comprehensiveness, participation, social inclusion, and continuity of care (Rodríguez, 2013). These components, even if they are not fully agreed in the different PCC conceptual models, may be considered central elements alongside the three principles previously identified (Smith and Williams, 2016).

A necessarily related issue is the measurement of PCC, which can vary according to whether multi-item or single-item measures are used (e.g., Rosenzveig et al., 2014). Measures also vary according to whether they include unresolved issues or are in a state of development. These unresolved issues stem from several problems that occur consistently in the measurement of PCC, such as the lack of clarity in the necessary quality indicators of these instruments, the absence of an empirically agreed conceptual structure, and the variety of instruments with differing psychometric qualities. For example, the most recent synthesis of research on PCC measurement in hospital centers reported a tendency for the instruments used to not fully include the proposed theoretical dimensions, as well as a frequent under-reporting of their psychometric properties (Handley et al., 2021).

On the other hand, in a study that examined the views of clinicians, quality evaluators and academics in the context of measuring PCC, the issues that emerged were, among others: the difficulty of measuring the subjectivity involved in the identification of the dimensions of the PCC; how to differentiate between the dimensions in practice; and the infrequent use of standardized measures (Ahmed et al., 2019). Another synthesis study identified the partial coverage regarding the dimensions that are considered key in the evaluation of PCC (Hudon et al., 2011), and the partial evidence obtained from single studies that investigate a narrow range of evidence for validity (Rosenzveig et al., 2014), as other characteristics of the current state of development of measures on PCC. Finally, the latent processes involved in the effectiveness of PCC, defined as moderating or mediating processes, are still a dark area of knowledge that interacts with the quality of the measurements (Rathert et al., 2013).

This may not come as a surprise regarding attributes that besides their conceptual complexity, such as the concordance of shared values between patients and the doctor (Winn et al., 2015), also exhibit high instrumental and methodological heterogeneity in their psychometric properties. Overall, there is a resulting difficulty in synthesizing research on a specific theoretical dimension of the PCC (Winn et al., 2015), which also seems to apply to the rest of the proposed theoretical dimensions of this approach.

Among the existing measures related to PCC, the *Person-Centered Care Assessment Tool* (P-CAT; Edvardsson et al., 2010) is an instrument designed in Australia to measure the PCC approach, and has gained wide acceptance in recent years (Martínez et al., 2015). It was developed based on research literature and interviews with professionals, experts in the field, people with dementia, and family members. It was mainly oriented toward long-term residential settings for the elderly. However, it has begun to be used in other settings, such as oncology units (Tamagawa et al., 2016) and psychiatric hospitals (degl'Innocenti et al., 2020). The tool consists of 13 items grouped into 3 subscales: personalized attention (7 items), organizational support (4 items), and accessibility of the environment (2 items). The items are ordinally scaled over 5 points (from “totally disagree” to “totally agree”); so that the possible total score ranges between 13 and 65, with the highest values being those that indicate a greater degree of attributes associated with caring for the person. In their original study (Edvardsson et al., 2010), the instrument showed satisfactory internal consistency for the total scale (α = 0.84), as well as good test-retest reliability (*r* = 0.66) over a time interval of 1 week.

From a practical point of view, the P-CAT is shorter and easier than other available tools, which makes it easy to apply and interpret, while at the same time capturing all the essential elements of PCC as described in the literature. Given the potential emic characteristics of this measure, the P-CAT has been adapted in several countries with wide cultural and linguistic differences, such as Norway (Rokstad et al., 2012), Sweden (Sjögren et al., 2012), China (Zhong and Lou, 2013), South Korea (Tak et al., 2015), and Spain (Martínez et al., 2015). However, the P-CAT test has been shown to have several weaknesses in its development, such as the impossibility of evaluating the validity criterion, and a poor internal consistency for the third subscale (α = 0.31; Edvardsson et al., 2010). Furthermore, in contrast to its wide range of use, no study has been conducted in which its mean reliability was established through formal procedures.

Estimating the mean reliability stems from the tradition of integrating research on a specific parameter, which is central to meta-analytic studies. Also called *reliability generalization*, this methodology facilitates the obtaining of a meta-analytic estimation of the reliability of the scores, whose integrity varies between the administrations, and studies the characteristics of the study that can better predict these variations (Vacha-Haase, 1998). Obtaining a meta-analytic parameter such as mean reliability is of key importance beyond its theoretical implications, since a practical implication is that allows to correctly estimate the size of the effect and the results of the statistical significance tests Wilkinson and APA Task Force on Statistical Inference (1999). On the other hand, a key theoretical implication is that mean reliability imposes limits on the interpretation of the measurement validity results (Feldt, 1997; Frary, 2000), a matter of general application that is deduced from the classical theory of tests (Feldt, 1997).

Applied to the P-CAT, the reliability of this test's scores can serve as important reference information for future studies, where the design of the sample size and the contextual conditions in which data are collected affect the quality of the study, and one of the fundamental indicators is the degree of random error in measurement (Berchtold, 2016). A meta-analytical approach to the reliability of the P-CAT not only aims at the estimation of overall reliability, but also at the investigation of its variability; for this reason, the choice of moderator variables is important insofar as they can explain part of the variability in the reliability coefficients. There are three groups of variables that can affect these coefficients (Sánchez-Meca et al., 2009): methodological factors (e.g., answer collection format, test version, group size, number of items), group origin and composition factors (e.g., clinical vs. normal nature, age and variability of the subjects, distribution by sex, ethnicity or educational level), and contextual factors (e.g., purpose of study, nationality of participants, year of study completion).

The objective of this study is to perform a reliability generalization meta-analysis to estimate the internal consistency of the P-CAT and analyze possible factors that may affect it. Additionally, a secondary objective is to evaluate the substantive or methodological characteristics of the studies that are statistically associated with the reliability coefficients, such as the year of publication, the continent of application, the version of the test (original, translation free, or adaptation), the form of application of the test (face-to-face or other, such as by telephone or internet), the context of care (geriatric residence or other), the sex of the participants, the mean age of the sample (and its standard deviation), and the mean score obtained in the test (and its standard deviation). This information is useful in order to understand, through quantitative data, which variables can affect the reliability of the instrument; and consequently, to offer guidelines to researchers and healthcare professionals to determine in what type of sample and contexts the P-CAT tends to produce more reliable scores.

## Methods

### Procedure

This study includes a reliability generalization meta-analysis of the P-CAT. The procedure followed is divided into two steps. First, a systematic review was carried out following the PRISMA methodology (Urrútia and Bonfill, 2010). A meta-analysis was then carried out following the recommendations of the REGEMA guidelines (Sánchez-Meca et al., 2021). We also followed specific guidelines for performing reliability generalization meta-analyses (Sánchez-Meca et al., 2009; Rubio-Aparicio et al., 2018).

### Search

Initially, a search was carried out in the Cochrane database to find meta-analyses or systematic reviews carried out on the P-CAT. Since none were found, we then searched the Web of Science, PubMed, and Scopus databases. These databases are the main sources of published articles that have passed through high-quality editorial processes and content review (Falagas et al., 2008). As a search formula, the original P-CAT article (Edvardsson et al., 2010) was located, and all those articles that cited it were identified and analyzed. A complementary search was also carried out in Google Scholar so as to include “gray” literature, thus reducing the effects of publication bias (Molina, 2018). Finally, the references of the included articles were reviewed in order to collect other articles that met the search criteria but were not present in any of the aforementioned databases.

### Elegibility Criteria

Inclusion and exclusion criteria were used.

#### Inclusion Criteria

Articles had to meet a series of inclusion criteria to be incorporated into the meta-analysis: (a) be experimental or quasi-experimental studies; (b) apply the P-CAT; (c) present a sample composed of professional caregivers; (d) provide information on the reliability of the instrument in their sample(s) through the coefficient of α; (e) inform about the sample size (N); and (f) allow access to the full text of the article. No range of years was imposed since all articles citing the P-CAT were searched and analyzed.

#### Exclusion Criteria

On the other hand, those investigations that presented at least one of the following exclusion criteria were discarded: (a) not being experimental or quasi-experimental studies; (b) not applying the P-CAT; (c) not reporting the reliability of the instrument, or reporting reliability only through values cited from previous research; (d) not indicating the sample size (*N*); or (e) presenting a duplicate sample with other articles. In case (e), only the oldest article was selected, or the oldest one that provided the α coefficient of the total score and not of each subscale (if the oldest article did not do that), and the rest were discarded.

### Study Selection

The search was conducted in February 2021 by a single researcher. The same researcher then screened the 106 selected articles by reading the abstracts (after eliminating 122 duplicate articles in the various databases).Only 27 articles were considered adequate after undergoing the initial screening process. After that, the same researcher performed a full analysis of the body text of the articles to identify whether they met the exclusion criteria, and as a result 5 of these 27 articles were eliminated. Finally, he checked the references of the included articles. An article found in the references of one of the selected studies was included, resulting in a final total 23 articles that met the inclusion criteria being selected to carry out the systematic review.

**Figure 1:**
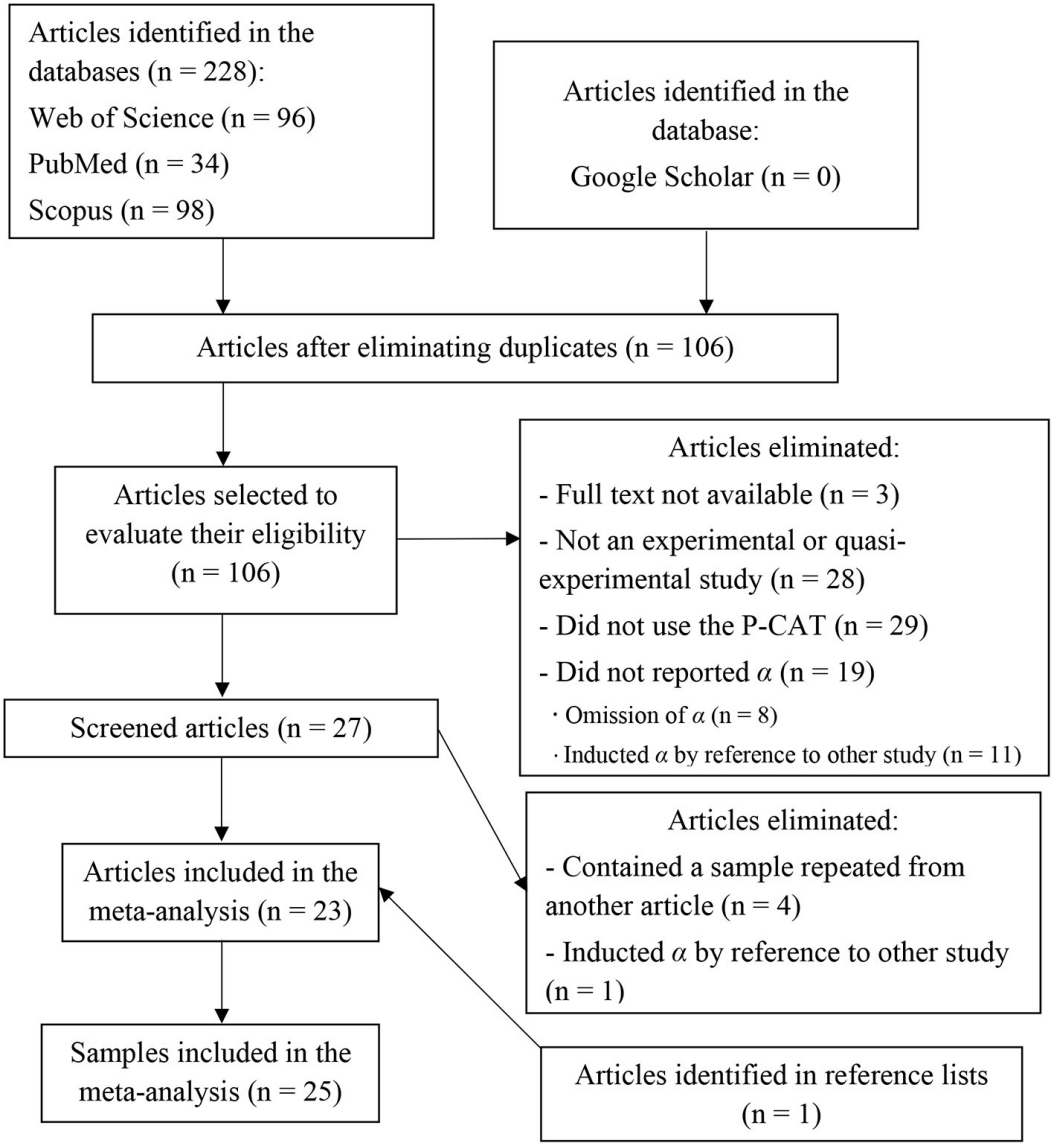
Flowchart of the screening and selection process for the articles in the meta-analysis.

In longitudinal studies, or others that included more than one measurement performed on the same participants, the first study was selected. The cases in which the α coefficient was reported for each of the subscales, and not for the total scale, were regarded as two different articles with their corresponding samples. In Figure 1 the selection and screening process of the articles are illustrated in detail.

### Data Extraction

The α coefficient (or coefficients in those articles that presented the α of the subscales) was extracted from all the selected studies. Two types of studies were found in which the own α was not reported: α not reported by omission (i.e., nothing was indicated about reliability in the study) and α by induction (i.e., reported by reference to another study). The number of studies found that did not report the own alpha was 20 (8 by omission and 12 by induction). No other internal consistency coefficients (e.g., omega) were found. Given the predominant use of the P-CAT total score in psychometric and non-psychometric studies, the α coefficient of the P-CAT will be extracted and meta-analyzed.

Likewise, the descriptive values of variables from all the selected articles were coded, so as to subsequently evaluate their effect on the homogeneity of the reliability coefficients. The coded variables were: (a) continent in which the P-CAT was applied; (b) year of publication of the article; (c) whether the test was used in its original version, free translation or adaptation to another language; (d) the method of application of the test (coded as face-to-face or other); (e) the environment in which professional care was carried out (coded as geriatric residence or other); (f) the sex of participants (coded as number of women and number of men); (g) the mean and standard deviation of the age of the participants; and (h) the mean and standard deviation of the P-CAT scores in the study sample.

The relevance of these variables comes from their typical use as reported in the literature; that is, for their selection, indications proposed in guidelines for the performance of reliability generalization meta-analysis were followed (Henson and Thompson, 2002), and previous reliability generalization studies were also followed as examples (Sánchez-Meca et al., 2016). Sociodemographic variables such as gender and age of the participants were selected since they have been typically used in the literature to predict the variance of reliability in generalization studies. Likewise, due to the wide range of use of the P-CAT instrument and the potential emic characteristics of the measure, variables such as the continent of application and the adaptation or translation to another language were coded in order to quantify possible variations in reliability due to cultural differences. Variables such as the mean and deviation of the scores were also taken into account to verify their effect because, as psychometric theory points out, there is a positive correlation between the variability of the scores and the reliability exhibited by the sample in question (Sánchez-Meca et al., 2016). In addition, since the P-CAT has begun to be applied in care contexts other than the one proposed by the authors in the study in which it was developed, this variable has been selected to check if this change in the care environment affects the reliability of the care quality instrument. Lastly, it was verified whether the method of application of the instrument in a way other than the traditional one (face-to-face), such as over the internet, can affect reliability.

### Statistical Analysis

First, to assess publication bias, the Egger test was used, the null hypothesis of which was that there was no publication bias in the sample of selected articles. Second, Cochrane's *Q* statistic was used to evaluate the homogeneity of the reliability coefficients, the null hypothesis of this test being that there was no homogeneity in the reliability coefficients of the sample of selected studies. This was complemented with the *I*^2^ index (Higgins and Thompson, 2002), which is a measure of the degree of heterogeneity of the reliability coefficients.

Regarding the index used, this was the α coefficient. One of the essential requirements to carry out a meta-analysis is that the scores (in this case, the α value) follow a normal distribution (Sánchez-Meca and López-Pina, 2008). To achieve this, as a third step, the α values were transformed to *T-*values using the formula *T* = (1–α)^1/3^ (where α is the coefficient of the total score for each sample), and each transformed α was weighted with the inverse of the variance using the formula *T*_+_ = Σ_*i*_*w*_*i*_*T*_*i*_/Σ_*i*_*w*_*i*_. This weighting was done because the weighting factor that obtains the lowest error variance is the one obtained by calculating the inverse of the variance of the sampling distribution of the statistic in question (in this case, the *T* scores; Sánchez-Meca and López-Pina, 2008). Fourth, to calculate the weighted mean value of α (i.e., expressed as a weighted *T*-value), and conditional on the evaluation of heterogeneity, a random effects statistical model was assumed using the restricted maximum probability method (REML), and a 95% confidence interval was calculated for this value using the method proposed by Hartung and Knapp (2001).

Fifth, to estimate the influence of the moderating variables and the variance between studies, a mixed effects model was assumed using the REML. Likewise, the method improved by Knapp and Hartung (2003) was used to calculate the mean value of α and the statistical significance of each moderator, as recommended in other meta-analyses (e.g., Rubio-Aparicio et al., 2019). To determine the influence exerted by the moderating variables, each of them was analyzed in isolation. The continuous moderating variables were year of publication, number of women, number of men, mean age and standard deviation of the age of the participants, and the mean and standard deviation of the scores in the study sample. The categorical moderating variables were continent of application, test version, administration method, and care context. For the continuous moderators, a series of simple linear meta-regressions were performed using α as the dependent variable, while for the categorical moderators, a series of weighted ANOVAS were performed. For all the analyses performed, version 2.1.0 of the R *Metafor* package (Viechtbauer, 2010) was used.

### Corroboration of the Meta-Analytical Report

To verify that the present work has been carried out according to the indications of REGEMA, a self-analysis was carried out in which the checklist proposed by this same guide was completed, visible in Appendix 2. It consists of 30 items that evaluate the most relevant points of each section (i.e., title, abstract, introduction, method, results, discussion, funding, and protocol), by means of categorical answers “yes” or “no” according to whether it meets the proposed item or not, respectively. The possibility “not applicable” is offered, in case the item is not relevant for this study. In order to facilitate the search for the answers offered, the page in which each item was located was pointed out.

## Results

### Evaluation of Selection Bias

**Figure 2:**
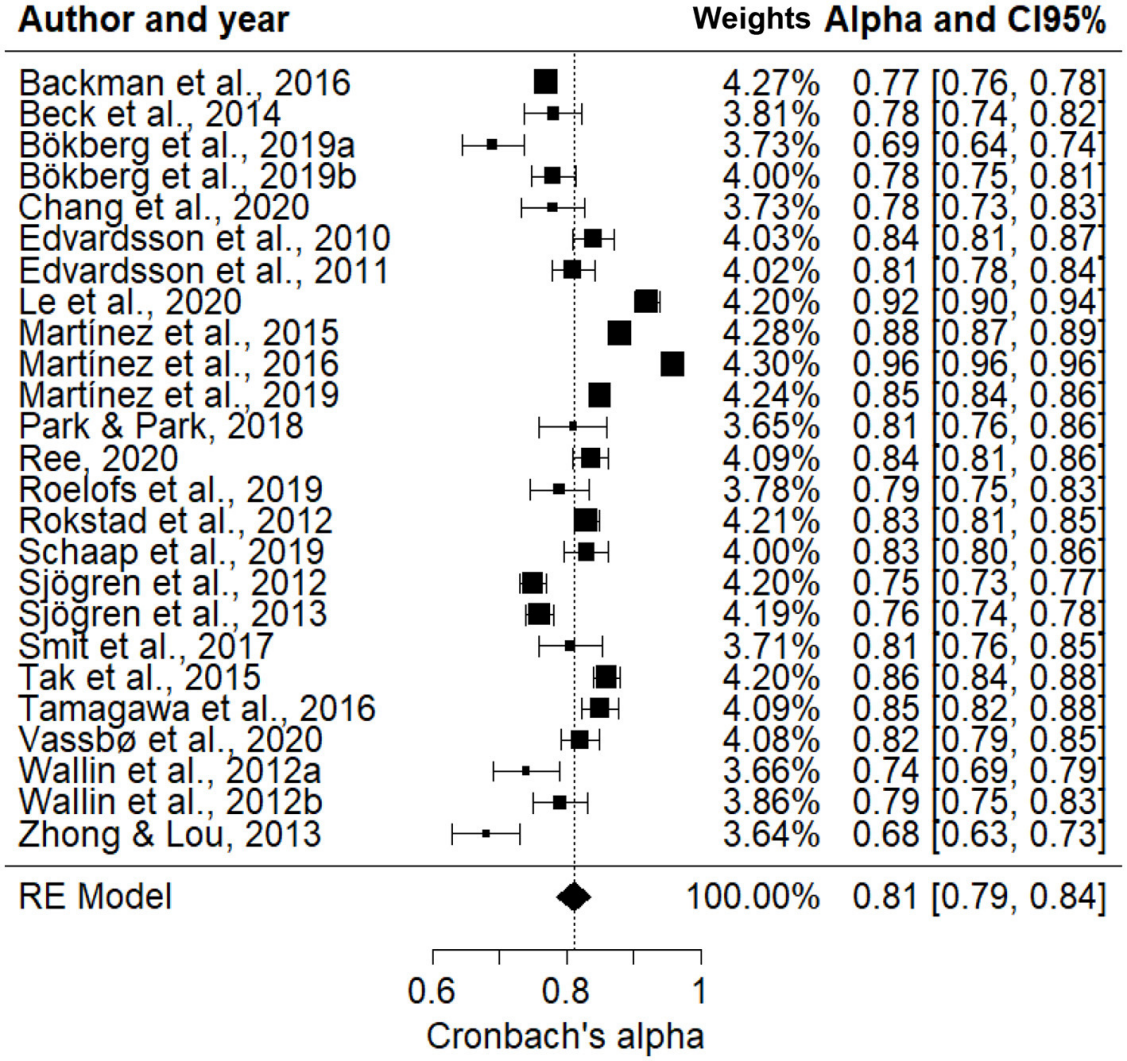
Forest plot with weighted values of α.

The total number of participants collected in the meta-analysis of the 25 selected samples was 15,149. The first analysis performed was the Egger test to detect the presence of a possible selection bias. The results of the test provided no evidence for the presence of this bias [*t*_(23)_ = −0.0503, *p* = 0.9599]. The mean value of α for the 25 meta-analysis samples was 0.81 (95% CI: 0.79, 0.84). Figure 2 shows the weighted value of α for each of the samples analyzed, as well as the 95% confidence intervals and sample size.

It was observed that 12 studies (48%) obtained α coefficients with greater distance from the central tendency (e.g., Zhong and Lou, 2013; Bökberg et al., 2019; Le et al., 2020). On the other hand, the studies with less weight, and consequently with a greater variation due to the size of their samples, tended to be located below the meta-analytic alpha value, suggesting a possible restriction of the variance that commonly occurs.

### Evaluation of Homogeneity

The results reflected heterogeneity in the sample, *Q*_(25)_ = 204.64, *p* < 0.0001. The *I*^2^ index yielded a proportion of variability attributable to heterogeneity of 85.83%, a value considered high. Given the heterogeneity of the studies, the next step was to analyze the moderating variables to see to what extent they affected the homogeneity of the reliability coefficients. In this analysis, the α values (or more precisely, their transformed *T*-values) took the role of the dependent variable (DV), while the rest of the variables collected in the studies become the independent variables (IVs).

### Evaluation of the Moderators

The results of the simple linear meta-regression to analyze the association between the different continuous IVs and the DV are shown in Table 1. The variables that independently explained most proportion of the variance were the mean P-CAT score with 85.99%, followed by age with 38.98%, and deviation in age with 8.18%. However, the only variable that presented a statistically significant relationship with the α coefficient was mean age. To examine the relationship between mean age and the reliability coefficient, a Pearson correlation was performed. A high level of negative linear association was observed (*r* = −0.62, *p* = 0.003).

**Table 1:** Analysis of the continuous moderator variables.

**IV (*k*)**	***b***	**CI 95% *b***	***p***	***F***	***Q_***E***_***	**% *R^**2**^***
Year of publication (25)	−0.002	−0.008–0.003	0.41	0.69	196.93^***^	0
Women (22)	0	−0.0–0.0	0.86	0.03	192.28^***^	0
Men (22)	−0.0001	−0.0006–0.0003	0.60	0.30	199.01^***^	0
Age (mean) (21)	0.007	0.003–0.011	0.003^**^	11.79^**^	89.03^***^	38.98
Age (SD) (17)	0.014	−0.005–0.032	0.14	3.94	139.85^***^	8.18
Score (mean) (5)	0.006	0.001–0.013	0.08	6.77	3.94	85.99
Score (SD) (4)	0.010	−0.057–0.077	0.59	0.41	9.86^**^	0

*k, number of samples; b, regression coefficient of the moderator variable; F, statistic of the significance test for the moderator variable; Q_E_, statistic of the test whether the model is well-specified; R^2^, proportion of the variance explained by the moderator variable*.

** *p < 0.001*,

*** *p < 0.0001*.

Next, to analyze the relationship between the categorical IVs and the DV, a series of weighted ANOVAS were performed. Table 2 shows the results, showing which of the IVs were significantly related to the α coefficient. None of the categorical variables presented statistically significant results. Furthermore, the percentage of the variance explained was 0% in all cases.

**Table 2:** Analysis of the categorical moderator variables.

**IV levels (*k*)**	***b***	**CI 95% *b***	***p***	***F***	***Q_***E***_***	**% *R^**2**^***
Continent (25)				0.11	202.83^***^	0%
Asia (5)	0.008	−0.07–0.09	0.83			
Europe (17)	0.009	−0.06–0.08	0.80			
Americas (1)	−0.016	−0.13–0.10	0.78			
Oceania (2)	0.016	−0.10–0.13	0.78			
Version (25)				0.13	203.40^***^	0%
Original version (3)	−0.014	−0.09–0.06	0.70			
Free translation (3)	0.015	−0.06–0.09	0.70			
Validated version (19)	0.014	−0.04–0.07	0.61			
Method of administration (25)				0.02	204.45^***^	0%
Face-to-face (7)	0.003	−0.04–0.04	0.89			
Other (18)	−0.003	−0.04–0.04	0.89			
Context of care (25)				0.97	201.62^***^	0%
Geriatric residence (19)	0.020	−0.02–0.06	0.34			
Other (6)	−0.020	−0.06–0.02	0.34			

*k, number of samples; b, regression coefficient of the moderator variable; F, statistic of the significance test for the moderator variable; Q_E_, statistic of the test whether the model is well-specified; R^2^, proportion of the variance explained by the moderator variable; IV, independent variable*.

*** *p < 0.0001*.

## Discussion

Traditionally in the literature, reliability has been used to refer to the reliability coefficients of classical test theory (i.e., the correlation between scores in two equivalent forms of tests; American Educational Research Association, 2014). It has also been used to refer to the consistency of scores in replicates of a test procedure, regardless of how this consistency is estimated or reported (Bökberg et al., 2019). In this sense, reliability is not an inherent property of the test, but depends on scores in a test for a particular population (Wilkinson and APA Task Force on Statistical Inference, 1999), and their variability between samples is a realist presumption. In the current study we look for meta-analysis of internal consistency (i.e., α coefficient) of P-CAT, and a mean α value equal to 0.81 was observed, meta-analyzed from a total of 23 articles that included 25 samples (*N*_total_ = 15,149). This magnitude of the α coefficient is considered good based on some arbitrary classifications (Ponterotto and Ruckdeschel, 2007; Vaske et al., 2018) and, accordingly, the scores suggested for basic research (Nunnally and Bernstein, 1994).

However, qualification of the reliability of the P-CAT scores must be framed in terms of their intended use, and the decisions that influence their users. The P-CAT is used for research, and its use has been extended toward the characterization of psychosocial factors in the caregiving role, and within a practical, brief and efficient use orientation. Therefore, considering a rationally constructed three-way matrix (Ponterotto and Ruckdeschel, 2007), based on the magnitude of the coefficient, the sample size, and the number of total score items, the level can be considered minimally acceptable, a level that is similar to 9 arbitrary rating sources cited by Ponterotto and Ruckdeschel (2007; Table 1) for measures used in psychology research. Similarly, in a review of test reviews, journal articles, and manuals (Charter, 2003), the meta-analytic reliability of the P-CAT can be placed at a level at the median of instruments (Table 2, “others” test; Charter, 2003), 0.81.

These results indicate that the P-CAT gives acceptably consistent scores when its use is oriented to the description and investigation of groups; in contrast, for making individualized decisions for patients, the amount of error around the score does not guarantee high sensitivity to detect a change in attitudes to care on an individualized basis. With 95% confidence, the mean α, however, can be as low as 0.79 in the population, indicating increasing error variance. We should note that general interpretation based on arbitrary classifications is not without controversies: for example, Taber (2018) found 18 variations in the labels used to classify the size of the α coefficient, as well as a clear discrepancy in delimiting one classification from another. These levels of acceptability can be understood as connected to several misconceptions about the use and interpretation of α (Ponterotto and Ruckdeschel, 2007; Cho and Kim, 2015). Some updated proposals based on modeling (e.g., Cho, 2016) or those derived from solid theoretical principles (e.g., Ponterotto and Charter, 2009) may be options that each individual study should take into account.

The heterogeneity of the reliability in this study is close to 85%, with values over 75% generally considered high (Molina, 2018). This magnitude implies that there are study conditions that increase variability, with an index so high that it was necessary to carry out an exhaustive analysis of the moderator variables that may affect it. Indeed, in the first place, after the analysis of the continuous moderators, it was observed that the reliability of the P-CAT is not affected by the year of publication. Nor does participant sex seem to influence reliability, since the instrument was developed to assess PCC by caregivers without taking patient sex into account, meaning it is important that it has a good consistency regardless of this characteristic. This suggests that the P-CAT can yield comparable scores precision in the perception of male and female patients, and one implication is that the client-centered clinical intervention environment could be equally expressed in patients, regardless of their sex. However, this statement is conditioned by the assumption of equivalence of measurement between the two groups.

In the analyses, it was observed that only the mean age of the participants was related to the reliability of the instrument, with a considerable proportion of explained variance. Specifically, the mean age showed a negative and statistically significant correlation with the reliability coefficient, which means that the samples with younger participants exhibited better average reliability than the samples with older participants. This result suggests that the P-CAT may be adequate as a general measure of PCC levels, and that the comparison between groups of participants of different ages requires considering the different error variance in the groups. Because the comparison of groups requires the invariance of the measurement parameters (for example, configuration, factor loadings, etc.), it cannot be stated whether the heterogeneous reliability reflects the lack of invariance between groups of different ages. This aspect must be resolved in specific validation studies, through SEM modeling, or via item response theory, by examining the possible differential functioning of the items in the test.

Second, when analyzing the categorical moderators, it was found that none of the categorical variables presented statistically significant results, with the proportion of the explained variance having a value of 0% in all cases. In relation to the cultural origin of the sample (i.e., continent of application), Asia, the Americas and Oceania had validated versions in some of their countries and languages with good psychometric properties, so neither of these two versions should influence the coefficient α. Only three studies in Europe used free translations, something that is currently discouraged (Sousa and Rojjanasrirat, 2011). However, in this case they had an α coefficient of around 0.8, considered good (Ponterotto and Ruckdeschel, 2007; Vaske et al., 2018), so this does not seem to have affected the reliability of the instrument.

Regarding the variables of method of administration and context of care, these did not yield statistically significant results, with the percentage of variance explained being effectively zero in both. This absence of differences is aligned with the trend toward the equivalence of measurement between evaluations applied online and in a traditional pencil-and-paper form (de Beuckelaer and Lievens, 2009). The implications of this are, firstly, that the P-CAT has proven to be reliable when applied in different ways, so that it can be used in research regardless of how the data is collected. Secondly, although the P-CAT was originally developed for nursing home settings, the use of the instrument in other types of settings does not seem to produce problems in the reliability variance, and the inclusion of studies in other types of care contexts (e.g., oncology centers or hospitals) does not affect the reliability of the instrument. This potential generalization of the use of the P-CAT to produce adequately reliable scores, however, is not evidence of the validity of its internal structure, and an argument in this regard is presented in the next paragraph.

Some complementary observations of the individual studies can serve as information aligned to the reliability reporting practices of the P-CAT. Specifically, it was rare to find corroboration of the dimensionality of the P-CAT scores, possibly influenced by the presumption of established dimensionality from the original study or subsequent validation studies. Given that the synthesis studies on the measurement of PCC have characterized it as a space where there is underreporting of psychometric properties and insufficient evidence of validity, substantive non-psychometric studies require providing evidence of the dimensionality of the scores, to validate the use of the α coefficient in particular (Savalei and Reise, 2019). This ensures that the reliability estimate is valid and adequate for the data (Cho, 2016), and avoids measurement validity induction from research carried out in different contexts, on qualitatively different samples, and with different study objectives (Merino-Soto and Calderón-de la Cruz, 2018; Merino-Soto and Angulo-Ramos, 2020, 2021). Part of this specific underreporting occurred in the interfactor correlations of the P-CAT, given that the psychometric studies that obtained a multidimensional factorial solution did not report this important psychometric parameter, which helps to diagnose the degree of dependence between factors and, consequently, the multidimensionality of the P-CAT.

Finally, and closely linked to the above, the P-CAT was created as a multidimensional measure, but the predominant use of the total score implies that users worked with the assumption of unidimensionality. Indeed, in about 13 substantive studies reviewed here, the total score was preferred over the individual dimension scores identified (e.g., Rokstad et al., 2012; Tak et al., 2015; Le et al., 2020). Also, Martínez et al. (2015) found that the multidimensional and unidimensional model were indistinguishable in their SEM fit indices, additionally with interfactor correlations >0.90. Therefore, the present study was oriented toward the reliability of the total score.

Regarding the limitations of the present study, firstly, the search was carried out only by one person, so an estimate of inter-rater reliability could not be made. Secondly, there were few articles found that used the P-CAT, partly due to its recent development; and even fewer that reported α for their own sample. In future research it would be interesting to analyze other psychometric properties of the P-CAT, such as validity, specificity or sensitivity.

In contrast to the above, one of the strengths of this study was to minimize the presence of biases that could alter the results. Indeed, to minimize publication bias, Google Scholar was included as one of the databases, thus trying to avoid excluding unpublished research from the search. Likewise, language bias was also reduced, by avoiding overrepresentation of studies in one language, and underrepresentation in others (Grégoire et al., 1995).

## Conclusion

Based on the results obtained in this study, the internal consistency of the P-CAT is not affected by continuous variables such as the year of publication, the number of participants of each sex, the age deviation, or the mean and standard deviation of the test scores. It also showed that neither the continent where the P-CAT was applied, nor the version of the test, nor the method of administration, nor the context of care seemed to affect the reliability of the instrument. In this study, only the variable of mean age was related to the reliability coefficient, obtaining a high level of negative linear association. It is suggested that the comparison between groups of participants of different ages requires considering the different error variance in the groups. Finally, the door is left open to research on the application of the P-CAT in settings other than geriatric residences, since the inclusion of studies with other types of care contexts did not affect the reliability of the instrument. In general, the results obtained in this study indicate that the P-CAT gives acceptably consistent scores when its use is oriented to the description and investigation of groups.

## Data Availability Statement

The original contributions presented in the study are included in the article/Supplementary Material, further inquiries can be directed to the corresponding author/s.

## Author Contributions

LB-L and MM-V: conception and design of the study. LB-L: data collection, management, and analysis. LB-L, MM-V, CM-S, and JL: manuscript critical review, editing, and approval. All authors contributed to the article and approved the submitted version.

## Funding

Funds for open access publication fee: National University Federico Villareal.

## Conflict of Interest

The authors declare that the research was conducted in the absence of any commercial or financial relationships that could be construed as a potential conflict of interest.

## Publisher's Note

All claims expressed in this article are solely those of the authors and do not necessarily represent those of their affiliated organizations, or those of the publisher, the editors and the reviewers. Any product that may be evaluated in this article, or claim that may be made by its manufacturer, is not guaranteed or endorsed by the publisher.
